# Isolated Lesser Trochanter Fractures in Adults: A Systematic Review of Malignant, Infectious, and Benign Etiologies

**DOI:** 10.7759/cureus.113785

**Published:** 2026-08-01

**Authors:** Max Deneroff, Ethan J Sack, Austin Khau, Zubair Mohammed, Chadi Ellaham, Jared W Nichols

**Affiliations:** 1 College of Osteopathic Medicine, Kansas City University, Joplin, USA; 2 Osteopathic Manipulative Medicine, Kansas City University, Joplin, USA

**Keywords:** avulsion fracture, lesser trochanter fracture, metastatic bone disease, orthopedic oncology, pathologic fracture, systematic review

## Abstract

Isolated lesser trochanter (LT) fractures are uncommon in adults and may be the first clue to an underlying pathologic process. In contrast to adolescent apophyseal avulsions, the same finding in a skeletally mature patient has been linked to metastatic disease, primary bone tumors, hematologic malignancy, infection, and, less often, benign traumatic, stress-related, osteoporotic, or metabolic causes. This review examined the malignant, infectious, and benign etiologies, diagnostic pathways, treatments, and outcomes that have been reported in adults aged 18 years or older presenting with a radiographically isolated LT fracture or avulsion fracture. PubMed, Embase, and Scopus were searched for English-language case reports and case series with extractable patient-level data, and one additional eligible report was identified during peer review. Twenty-eight studies involving 46 published patient cases were included. Metastatic solid malignancy was the most commonly reported etiology (27/46, 58.7%), followed by primary malignant bone or plasma-cell tumors (7/46, 15.2%) and benign/nonpathologic causes, including metabolic bone disease (8/46, 17.4%). Hematologic malignancy (2/46, 4.3%), infectious disease (1/46, 2.2%), and other tumor-like disease (1/46, 2.2%) were less common. Among the 36 malignant cases, malignancy was first recognized after the LT fracture in 25 cases (25/36, 69.4%; equivalent to 25/46, 54.3% of all included cases), was known before the fracture in eight, and was unclear or not reported in three cases. These values describe selected published cases and should not be interpreted as population prevalence. Atraumatic, low-energy, or radiographically suspicious adult isolated LT fractures warrant evaluation for underlying malignancy or infection, while metabolic bone disease should remain in the differential diagnosis​​​​​​.

## Introduction and background

The lesser trochanter (LT), the insertion site of the iliopsoas tendon, is an unusual site for an isolated fracture in skeletally mature patients. In adolescents, LT avulsion is typically an apophyseal injury related to forceful hip flexion or sports activity. After skeletal maturity, the same radiographic finding carries a different implication. Adult isolated LT fractures are rare, and the evidence base consists mainly of case reports and small case series rather than prospective studies or standardized treatment pathways [1]. This case-based literature is highly susceptible to publication and selection bias and cannot establish the true frequency of malignancy or other etiologies among unselected adults.

In adults, the concern is that a small avulsed LT fragment may be the first finding that brings an occult lesion to attention, and the published adult literature spans malignant, infectious, benign traumatic, stress-related, osteoporotic, and metabolic diagnoses [2-29]. A radiology teaching-file review from a specialist orthopedic oncology center found LT avulsion in 15 of 295 aggressive proximal femoral tumors, underscoring how subtle the sign can be; metastatic disease was most common, but myeloma, Ewing sarcoma, chondrosarcoma, non-Hodgkin lymphoma, and giant cell tumor were also represented [29]. In the case literature, patients commonly present with hip or groin pain after no trauma or a low-energy event. The initial radiograph may show only an apparently isolated fragment, while CT, MRI, bone scan, PET/CT, or biopsy later reveals proximal femoral tumor infiltration. Reported malignant causes include metastatic carcinoma from lung, breast, colon, renal, nasopharyngeal, bronchogenic, and other primary sites, as well as primary malignant bone tumors, plasma-cell neoplasms, hematologic malignancy, and tumor-like disorders [2,3,6-9,11-17,20-23,25-27,29].

That pattern does not mean every adult LT fracture is malignant. Published cases also include tuberculosis, stress-related injury, low-energy osteoporotic fractures in elderly patients, nonpathologic traumatic injuries, and renal osteodystrophy associated with end-stage renal disease [4,5,10,18,19,24,28]. Papineni et al. reported a 31-year-old adult with a radiographically isolated left LT avulsion fracture after minor trauma in the setting of renal osteodystrophy and secondary hyperparathyroidism [28]. Although bilateral olecranon avulsion fractures occurred concurrently, the LT fracture itself had no reported intertrochanteric, subtrochanteric, femoral neck, or shaft extension and therefore met the anatomical inclusion definition. For orthopedic surgeons, radiologists, and oncologists, the differential includes metastatic disease, primary bone tumor, infection, metabolic bone disease, benign avulsion, occult intertrochanteric extension, and mechanical instability.

The workup is part of the treatment decision, not a separate issue. Plain radiographs can identify the LT fragment, but they may miss marrow replacement, cortical destruction, soft-tissue extension, or the true length of proximal femoral involvement. This limitation matters when hip or groin pain is evaluated with a single anteroposterior pelvis or hip radiograph; the fragment may be easier to see than the underlying lesion. For adult atraumatic LT avulsion, MRI of the hip, plasma electrophoresis, and staging studies have been recommended to evaluate for primary or metastatic bone disease [29]. In practice, advanced imaging can define the lesion, guide biopsy, evaluate systemic disease, and help determine whether prophylactic stabilization or reconstruction is needed. When imaging suggests tumor infiltration or infection, tissue diagnosis is often the step that separates observation or protected weight bearing from biopsy, oncologic therapy, radiation, intramedullary fixation, dynamic hip screw fixation, resection, arthroplasty, or endoprosthetic reconstruction [1-3,14,18,21,29].

To be clinically useful, a synthesis of this literature must do more than report how often malignancy appears in published cases. The primary review question was: among adults aged 18 years or older with a radiographically isolated LT fracture or avulsion fracture, what malignant, infectious, and benign etiologies, diagnostic pathways, treatments, and outcomes have been reported? The review therefore mapped the etiologic spectrum, described the diagnostic pathways reported in published cases, identified how often malignancy was recognized before or after the fracture, and connected diagnosis with treatment and structural risk.

## Review

Methods

Search Strategy and Study Selection

This descriptive systematic review was reported in accordance with PRISMA 2020 guidance [30]. A structured search was performed in PubMed, Embase, and Scopus from May 1, 2026, through July 15, 2026, with the final search in each database completed on July 15, 2026. Searches used database-specific combinations of terms for the LT and fracture or avulsion, and raw record counts were documented before duplicate removal. The search was limited to English-language full-text reports because translation resources were unavailable; the potential for language bias was considered when interpreting the findings. The combined search yielded 204 records: PubMed (n = 127), Embase (n = 32), and Scopus (n = 45). Complete database-specific search strategies are provided in Table 1. The review protocol was not prospectively registered. Eligibility criteria, extraction variables, etiologic categories, and the narrative synthesis plan were established before study screening and data extraction. The PRISMA flow diagram was generated using the PRISMA2020 R package and Shiny application (R Foundation for Statistical Computing, Vienna, Austria) [31].

**Table 1:** Database-specific search strategies and identified records The combined search identified 204 records before deduplication: PubMed (n = 127), Embase (n = 32), and Scopus (n = 45). LT: Lesser trochanter.

Database	Search strategy	Limits/screening rule	Records identified
PubMed	("lesser trochanter"[Title/Abstract] OR "lesser trochanteric"[Title/Abstract] OR "trochanter minor"[Title/Abstract]) AND (fracture*[Title/Abstract] OR avulsion*[Title/Abstract] OR "pathologic fracture"[Title/Abstract] OR "pathological fracture"[Title/Abstract] OR "Femoral Fractures"[Mesh] OR "Avulsion Fractures"[Mesh])	English; humans; adult eligibility applied during screening	127
Embase	('lesser trochanter':ti,ab,kw OR 'lesser trochanteric':ti,ab,kw OR 'trochanter minor':ti,ab,kw) AND (fracture*:ti,ab,kw OR avulsion*:ti,ab,kw OR 'pathologic fracture':ti,ab,kw OR 'pathological fracture':ti,ab,kw OR 'femur fracture'/exp OR 'avulsion fracture'/exp OR 'pathologic fracture'/exp)	Humans; English; adult eligibility applied during screening	32
Scopus	TITLE-ABS-KEY(("lesser trochanter" OR "lesser trochanteric" OR "trochanter minor") AND (fracture* OR avulsion* OR "pathologic fracture" OR "pathological fracture"))	English; adult eligibility applied during screening	45
Total	PubMed + Embase + Scopus	Total records before deduplication	204

**Figure 1:**
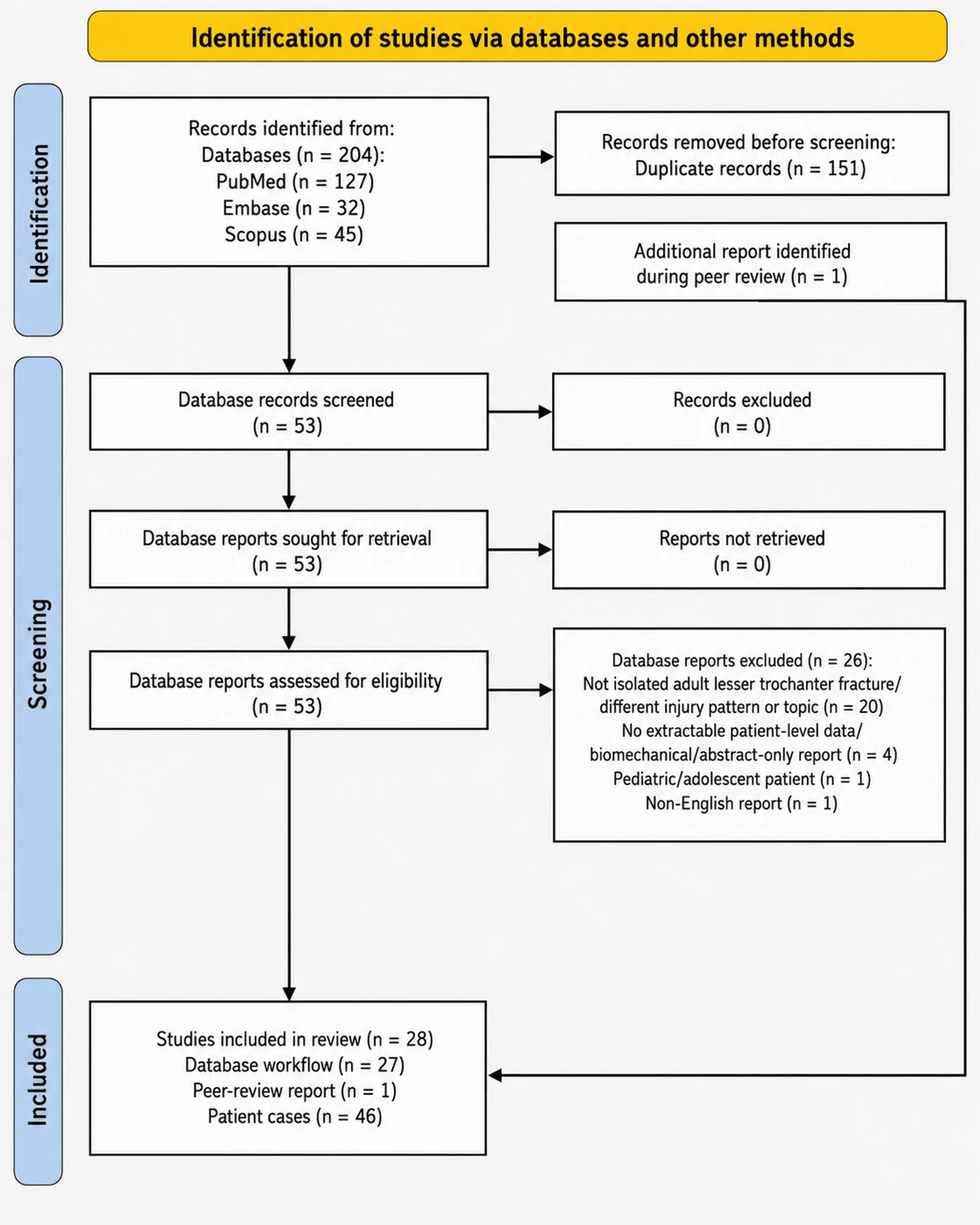
PRISMA flow diagram Flow of records through the systematic review process. Database searches included PubMed, Embase, and Scopus. One additional eligible report was identified during peer review and assessed under the same prespecified criteria. PRISMA: Preferred Reporting Items for Systematic Reviews and Meta-Analyses.

All retrieved citations were imported into Zotero (Corporation for Digital Scholarship, Vienna, VA) for citation management and deduplication. Potential duplicates were identified electronically and then manually verified by comparing titles, authors, publication years, and available DOI or PMID information. After removal of 151 duplicate records, 53 unique records remained. Titles and abstracts were screened independently by two reviewers, followed by independent full-text review of potentially eligible reports. Disagreements regarding eligibility were resolved through discussion and consensus. As every unique record contained potentially relevant LT fracture terminology, no record was excluded at the title/abstract stage, and all 53 reports advanced to full-text assessment; this accounts for the zero title/abstract exclusions shown in Figure 1.

Both reviewers also examined the reference lists of all included studies and relevant review-style publications and performed forward citation searching. Supplementary citation searching was completed on July 15, 2026, and identified no additional eligible original cases. During peer review, one potentially eligible report was identified through other methods, assessed against the same prespecified eligibility criteria, and included in the final review.

Studies were eligible if they reported adults aged 18 years or older with an isolated LT fracture or avulsion fracture and included extractable patient-level data. An isolated fracture was operationally defined as a discrete fracture fragment arising from the LT without reported intertrochanteric, subtrochanteric, femoral neck, femoral shaft, periprosthetic, or multifragmentary proximal femoral extension on the available radiographs or cross-sectional imaging. Concomitant fractures outside the proximal femur did not preclude inclusion when the LT fracture itself met this anatomical definition. CT or MRI findings were used to exclude occult extension when those studies were reported; later progression to a larger proximal femoral fracture did not negate eligibility if the initial presentation was isolated. Each included case had sufficient information to establish adult status, the isolated fracture pattern, and a final etiology, together with at least one extractable variable related to presentation, diagnostic evaluation, management, or outcome. Missing fields were recorded as not reported. Review articles, teaching-file reviews, and aggregate imaging summaries were used for reference-list checking and contextual discussion only unless they contributed original or clearly new cases. To identify overlapping cases and prevent double-counting, reports were compared by author group, institution, publication period, patient age and sex, tumor type, and clinical chronology; the most complete primary report was retained, and previously published cases repeated in review-style articles were not re-extracted. Inclusion and exclusion criteria are summarized in Table 2.

**Table 2:** Inclusion and exclusion criteria LT: Lesser trochanter.

Criterion type	Criterion
Inclusion	Patient aged 18 years or older
	Radiographically isolated lesser trochanter fracture or avulsion fracture
	Concomitant fractures outside the proximal femur permitted when the lesser trochanter fracture itself met the radiographic isolation definition
	Case report, case series, or observational report
	Extractable patient-level data establishing final etiology plus at least one variable on presentation, diagnostic evaluation, management, or outcome
	English-language full-text report
Exclusion	Patient younger than 18 years
	Adolescent athletic apophyseal avulsion injury
	Intertrochanteric fracture with lesser trochanter involvement
	Subtrochanteric fracture with lesser trochanter involvement
	Femoral neck fracture with lesser trochanter involvement
	Femoral shaft fracture with lesser trochanter involvement
	Periprosthetic fracture
	Multifragmentary proximal femur fracture in which the lesser trochanter was not the primary isolated fracture finding
	Biomechanical study without patient-level clinical data
	Abstract-only report
	Report without adequate patient-level details
	Non-English report
	Review article, teaching-file review, or aggregate imaging summary without original extractable cases

Data Extraction and Quality Appraisal

Extracted variables included author/year, patient age and sex, mechanism or trigger, presenting symptoms, fracture site/laterality, etiology group, final diagnosis or primary tumor, whether malignancy was known before the LT fracture, imaging/workup, LT/proximal femoral lesion tissue diagnosis or pathology confirmation, treatment, and outcome/follow-up. Two reviewers independently performed patient-level data extraction and quality appraisal, with disagreements resolved through discussion and consensus. The 22 case reports were appraised with the Joanna Briggs Institute (JBI) Critical Appraisal Checklist for Case Reports, while the six case series were evaluated with the dedicated JBI Critical Appraisal Checklist for Case Series, as described in the JBI Manual for Evidence Synthesis [32]. Appraisal findings were used to judge completeness, selection bias, and confidence in interpretation; studies were not excluded solely based on appraisal results (Table 3).

**Table 3:** JBI critical appraisal summary by study design Case-report domains: Q1 demographics; Q2 history/timeline; Q3 clinical condition; Q4 diagnostic methods/results; Q5 intervention; Q6 post-intervention condition; Q7 adverse events/harms; Q8 takeaway lessons. Case-series domains: Q1 clear inclusion criteria; Q2 condition measured in a standard/reliable way; Q3 valid identification methods; Q4 consecutive inclusion; Q5 complete inclusion; Q6 demographics; Q7 clinical information; Q8 outcomes/follow-up; Q9 reporting of site/demographic characteristics; Q10 appropriate statistical analysis. CR: Case report; CS: Case series; Y: Yes; N: No; U: Unclear; NA: Not applicable; JBI: Joanna Briggs Institute.

Studies (design)	Q1	Q2	Q3	Q4	Q5	Q6	Q7	Q8	Q9	Q10
Cho et al., 2020 [1] (CS)	Y	Y	Y	U	U	Y	Y	N	Y	Y
Herren et al., 2015 [2] (CR)	Y	Y	Y	Y	Y	Y	N	Y	NA	NA
Afra et al., 1999 [3] (CS)	Y	Y	Y	U	U	Y	Y	Y	Y	NA
Miyake et al., 2022 [4] (CR)	Y	Y	Y	Y	Y	Y	N	Y	NA	NA
Agrawal et al., 2023 [5] (CR)	Y	Y	Y	Y	Y	Y	Y	Y	NA	NA
Yu et al., 2024 [6] (CR)	Y	Y	Y	Y	Y	Y	N	Y	NA	NA
Creissen et al., 2020 [7] (CR)	Y	Y	Y	Y	Y	Y	N	Y	NA	NA
Kumar et al., 2017 [8] (CR)	Y	Y	Y	Y	Y	Y	N	Y	NA	NA
Pang et al., 2025 [9] (CR)	Y	Y	Y	Y	Y	Y	Y	Y	NA	NA
Sharma et al., 2022 [10] (CR)	Y	Y	Y	Y	Y	Y	N	Y	NA	NA
Vanhoenacker et al., 2021 [11] (CR)	Y	Y	Y	Y	Y	Y	N	Y	NA	NA
Uddin et al., 2016 [12] (CR)	Y	Y	Y	Y	Y	Y	N	Y	NA	NA
Rouvillain et al., 2011 [13] (CR)	Y	Y	Y	Y	Y	Y	N	Y	NA	NA
Khoury et al., 1998 [14] (CS)	U	Y	Y	U	U	Y	Y	N	Y	NA
Fox et al., 2014 [15] (CR)	Y	Y	Y	Y	Y	Y	N	Y	NA	NA
McCambridge et al., 2023 [16] (CR)	Y	Y	Y	Y	Y	Y	N	Y	NA	NA
Edmonds et al., 2003 [17] (CR)	Y	Y	Y	Y	N	N	N	Y	NA	NA
Bonshahi et al., 2004 [18] (CS)	Y	Y	Y	U	U	Y	Y	Y	Y	NA
Singh et al., 2015 [19] (CR)	Y	Y	Y	Y	Y	Y	N	Y	NA	NA
Bertin et al., 1984 [20] (CS)	Y	Y	Y	U	U	Y	Y	Y	Y	NA
Heiney and Leeson, 2009 [21] (CR)	Y	Y	Y	Y	Y	Y	Y	Y	NA	NA
Reategui-Villegas et al., 2013 [22] (CR)	Y	Y	Y	Y	Y	Y	Y	Y	NA	NA
Phan and Vu, 2018 [23] (CR)	Y	Y	Y	Y	Y	N	N	Y	NA	NA
Uzun et al., 2016 [24] (CR)	Y	Y	Y	Y	Y	Y	N	Y	NA	NA
Koçer et al., 2021 [25] (CR)	Y	Y	Y	Y	Y	Y	N	Y	NA	NA
Phillips et al., 1988 [26] (CS)	Y	Y	Y	U	U	Y	Y	N	Y	NA
Gradwohl and Mailliard, 1987 [27] (CR)	Y	Y	Y	Y	N	N	N	Y	NA	NA
Papineni et al., 2025 [28] (CR)	Y	Y	Y	Y	Y	N	N	Y	NA	NA

The etiologic groups (metastatic solid malignancy, primary malignant bone or plasma-cell tumor, hematologic malignancy, infectious disease, other neoplastic/tumor-like disease, and benign/nonpathologic cause) and the narrative synthesis methods were defined before data extraction. Meta-analysis was not performed because the evidence base consisted predominantly of case reports and small case series with heterogeneous clinical reporting. Results are presented descriptively using patient-level counts and clearly stated denominators. Details not explicitly reported were coded as not reported and were not assumed to be absent. Percentages represent reporting frequencies in selected published cases and were not interpreted as prevalence, diagnostic accuracy, comparative effectiveness, or recommended test utilization.

Results

Study Selection and Patient Characteristics

Twenty-eight English-language studies met inclusion criteria and contributed 46 original patient cases. The evidence base comprised 22 case reports and six case series; no comparative or prospective observational studies met inclusion criteria. Twenty-seven studies were identified from the database and citation-search workflow, and one additional eligible case report was identified during peer review and assessed using the same prespecified criteria. All included cases provided extractable adult and isolated-fracture status, a final etiology, and at least one patient-level variable related to presentation, diagnostic evaluation, management, or outcome. Table 4 summarizes the patient-level characteristics and outcomes, and Figure 1 shows the study selection process.

**Table 4:** Patient-level characteristics and outcomes of adult isolated lesser trochanter fractures Each row represents one extracted patient/case. Review-style articles contributed only original/new cases; prior literature-review cases were not re-extracted. The Papineni et al. [28] case was added during peer review and met the anatomical definition because the LT fracture itself was isolated within the proximal femur despite concomitant olecranon fractures. LT: Lesser trochanter; NR: Not reported; MUTARS: Modular Universal Tumor and Revision System; US: Ultrasound; IM: Intramedullary; ORIF: Open reduction and internal fixation; SFA: Superficial femoral artery; ABI: Ankle-brachial index; MRA; Magnetic resonance angiography; CTA: Computed tomography angiography; NSCLC: Non-small cell lung cancer; CECT: Contrast-enhanced computed tomography; ATT: Anti-tuberculosis treatment; PSA: Prostate-specific antigen; LCH: Langerhans cell histiocytosis; FDG: Fluorodeoxyglucose; DHS: Dynamic hip screw; SLR: Straight leg raise; NWB: Non-weight-bearing; GI: Gastrointestinal; TFN-A: Trochanteric fixation nail-advanced; PTH: Parathyroid hormone.

Study/report	Age/sex	Mechanism/presentation	Etiology group	Final diagnosis	Diagnostic workup	Management/outcome
Cho et al., 2020 [1]	Original case 1: 57/M	Individual mechanism NR; overall current series 5 no trauma/1 slip. Presentation: Groin pain/antalgic gait	Metastatic solid malignancy	Hepatocellular carcinoma	Radiographs; MRI; CT-guided core needle biopsy	Management: Tumor prosthesis/MUTARS. Outcome: NR
Cho et al., 2020 [1]	Original case 2: 68/M	Individual mechanism NR; overall current series 5 no trauma/1 slip. Presentation: Groin pain/antalgic gait	Metastatic solid malignancy	Thyroid cancer	Radiographs; MRI	Management: Tumor prosthesis/MUTARS. Outcome: NR
Cho et al., 2020 [1]	Original case 3: 56/M	Individual mechanism NR; overall current series 5 no trauma/1 slip. Presentation: Groin pain/antalgic gait	Metastatic solid malignancy	Renal cell carcinoma	Radiographs; MRI; CT-guided core needle biopsy	Management: Tumor prosthesis/MUTARS. Outcome: NR
Cho et al., 2020 [1]	Original case 4: 72/M	Individual mechanism NR; overall current series 5 no trauma/1 slip Presentation: Groin pain/antalgic gait	Metastatic solid malignancy	Prostate cancer	Radiographs; MRI	Management: Radiation therapy. Outcome: NR
Cho et al., 2020 [1]	Original case 5: 62/M	Individual mechanism NR; overall current series 5 no trauma/1 slip. Presentation: Groin pain/antalgic gait	Metastatic solid malignancy	Renal cell carcinoma	Radiographs; MRI	Management: Tumor prosthesis/MUTARS. Outcome: NR
Cho et al., 2020 [1]	Original case 6: 62/M	Individual mechanism NR; overall current series 5 no trauma/1 slip. Presentation: Groin pain/antalgic gait	Primary malignant bone/plasma-cell tumor	Osteosarcoma	Radiographs; MRI	Management: Bipolar hemiarthroplasty. Outcome: NR
Herren et al., 2015 [2]	61/F	Chiropractor treatment/minor manipulation. Presentation: Right groin pain, low back pain, weight loss, night sweats	Metastatic solid malignancy	Poorly differentiated breast carcinoma with liver/skeletal metastases	X-ray; CT pelvis; breast US/mammography; CT staging	Management: Conservative ortho care; palliative paclitaxel/bevacizumab; refused IM nail. Outcome: No complaints/no mobility limitation at 5 months
Afra et al., 1999 [3]	Case 1: 44/M	No antecedent trauma; acute worsening while sailing. Presentation: Left hip/groin pain; hip flexion weakness	Primary malignant bone/plasma-cell tumor	Solitary plasmacytoma	Radiographs; CT; technetium bone scan; skeletal survey	Management: Zickel nail + methylmethacrylate; postoperative irradiation. Outcome: Asymptomatic; reconstruction intact; no recurrence or progression to multiple myeloma at latest follow-up
Afra et al., 1999 [3]	Case 2: 69/M	No trauma; avulsion while exiting automobile. Presentation: Left groin pain, increasing pain/difficulty lifting limb	Primary malignant bone/plasma-cell tumor	Chondrosarcoma	Radiographs; MRI; bone scan; CT chest	Management: ORIF initially; wide en bloc proximal femur resection and custom proximal femoral endoprosthesis. Outcome: Local recurrence and pulmonary metastasis; died May 1991 after massive stroke with pulmonary metastases at death
Afra et al., 1999 [3]	Case 3: 19/M	No specific trauma; crack stepping onto curb. Presentation: Right groin pain; massive swelling of thigh/buttock	Primary malignant bone/plasma-cell tumor	Ewing sarcoma	Radiographs; CT	Management: Neoadjuvant chemotherapy/radiation; wide resection and custom proximal femoral component. Outcome: Widespread osseous/pulmonary metastases within 2 months; died 6 months later
Afra et al., 1999 [3]	Case 4: 36/M	No trauma stated; chronic back/radicular history with new groin pain. Presentation: Left groin pain and weak hip flexion	Primary malignant bone/plasma-cell tumor	High-grade chondrosarcoma	Radiographs; MRI	Management: Wide resection and allograft-prosthesis composite; later resection of metastatic nodules. Outcome: No additional local recurrence or metastatic disease for 2 years at latest follow-up
Miyake et al., 2022 [4]	40/M	Baseball pitching stress; stumble getting into car. Presentation: Gradual left hip pain with acute worsening	Benign/nonpathologic	Stress avulsion fracture	Radiographs; MRI; CT; gadolinium MRI; technetium scintigraphy	Management: Conservative. Outcome: Returned to sports 12 weeks; asymptomatic/full ROM at 1 year despite nonunion
Agrawal et al., 2023 [5]	19/M	Hockey injury with nondisplaced LT avulsion. Presentation: Intermittent claudication, calf cramping, medial knee numbness	Benign/nonpathologic	Adductor canal syndrome/SFA occlusion after LT avulsion	X-ray; duplex; ABI; MRA; angiogram; CTA	Management: SFA interposition bypass; later thrombolysis/angioplasty; aspirin/rivaroxaban. Outcome: Pain-free/without claudication at 4 months; not yet returned to hockey
Yu et al., 2024 [6]	71/M	No reported trauma. Presentation: Progressive left hip/thigh pain, difficulty weight bearing, weight loss	Metastatic solid malignancy	Metastatic paraganglioma	X-ray; MRI; labs; CT-guided biopsy; DOTATATE PET/CT	Management: Doxazosin; external beam radiation 3000 cGy/10 fractions. Outcome: Intermittent weight-bearing pain after RT; normetanephrine decreased at 3 months
Creissen et al., 2020 [7]	66/F	Atraumatic/popping sensation while sitting. Presentation: Right medial thigh/groin pain, bruising, antalgic gait	Metastatic solid malignancy	NSCLC lung carcinoma	X-ray; MRI; CT neck/chest/abdomen/pelvis	Management: Analgesia/non-weight bearing; afatinib palliative chemotherapy. Outcome: Good response with reduced tumor bulk at publication
Kumar et al., 2017 [8]	60/F	Atraumatic; getting out of bed. Presentation: Left hip pain with weight-bearing	Metastatic solid malignancy	Primary lung carcinoma with widespread metastasis	X-ray; MRI; CECT chest/abdomen; PET; proximal femur core needle biopsy	Management: Proximal femoral nail; chemoradiation/supportive care. Outcome: At home under supportive care at publication
Pang et al., 2025 [9]	79/M	Minor trauma/hip loading while bicycling. Presentation: Left hip pain and limited mobility; persistent pain after bed rest	Metastatic solid malignancy	Poorly differentiated squamous cell carcinoma of lung	X-ray; CT; MRI; contrast MRI; CT chest/abdomen; bone scan	Management: Initial bed rest; palliative radiotherapy; carboplatin/paclitaxel/tislelizumab. Outcome: Died of severe pulmonary infection
Sharma et al., 2022 [10]	40/M	Atraumatic; while climbing stairs. Presentation: Right groin pain, inability to bear weight	Infectious	Mycobacterium tuberculosis	X-ray; MRI; CT-guided biopsy; GeneXpert/cultures	Management: 4-drug ATT; staged weight-bearing/activity modification. Outcome: No pain/limitations at 2 years; displaced fragment persisted
Vanhoenacker et al., 2021 [11]	18/NR	No clear trauma; cyclist. Presentation: Months of left groin pain; night pain	Primary malignant bone/plasma-cell tumor	Ewing sarcoma	CT; MRI; follow-up radiograph; repeat MRI	Management: Neoadjuvant chemotherapy; resection; femoral prosthesis. Outcome: Uneventful follow-up
Uddin et al., 2016 [12]	48/F	Atraumatic/trivial movement getting into car. Presentation: Acute right hip pain; painful hip flexion	Metastatic solid malignancy	Undifferentiated bronchial adenocarcinoma	Radiographs; chest x-ray; CT chest/abdomen; MRI hip; MRI brain; bone scan; bronchoscopic biopsy; intraoperative femur biopsy	Management: Prophylactic cephalomedullary nailing; chemotherapy. Outcome: Transferred to oncology; longer outcome NR
Rouvillain et al., 2011 [13]	63/M	Atraumatic. Presentation: Thigh-root pain, limp, worsening function	Metastatic solid malignancy	Mucosecreting metastatic pulmonary adenocarcinoma	X-ray; MRI; CT; PET	Management: En-bloc resection/reconstruction with megaprosthesis; cisplatin/pemetrexed chemotherapy; T3 vertebral cementoplasty/radiation. Outcome: At 9 months, walking without cane/limp; normal pain-free hip mobility; PMA 18/18
Khoury et al., 1998 [14]	Case 1: 65/F	Non-traumatic. Presentation: LT avulsion due to neoplasm	Metastatic solid malignancy	Breast cancer metastasis	Details not extractable from available PDF	Management: Palliative radiation. Outcome: NR
Khoury et al., 1998 [14]	Case 2: 37/M	Non-traumatic. Presentation: LT avulsion due to neoplasm	Metastatic solid malignancy	Synovial sarcoma	Details not extractable from available PDF	Management: Prophylactic fixation. Outcome: NR
Khoury et al., 1998 [14]	Case 3: 49/M	Non-traumatic. Presentation: LT avulsion due to neoplasm	Primary malignant bone/plasma-cell tumor	Osteosarcoma	Details not extractable from available PDF	Management: Prophylactic fixation. Outcome: NR
Fox et al., 2014 [15]	75/M	Very low-impact fall while sitting. Presentation: Left groin pain and difficulty in weight-bearing	Metastatic solid malignancy	Renal cell carcinoma suspected/large renal mass with bony metastasis	Radiographs; CT hip/pelvis; CT abdomen/pelvis; chest x-ray; PSA	Management: Prophylactic IM nail; palliative chemotherapy/treatment. Outcome: Stable disease; discharged with palliative care
McCambridge et al., 2023 [16]	18/M	Pain after kicking punching bag; recurrence after baseball swing. Presentation: Right hip pain, night pain, lytic LT lesion	Other neoplastic/tumor-like	Multifocal single-system LCH of bone	Radiographs; MRI; bone scan; CT chest; FDG-PET/CT; brain imaging	Management: Prophylactic IM nail; curettage/cryoablation; vinblastine/prednisone. Outcome: Lesions improved after 3 months; chemotherapy stopped; continued oncology follow-up planned every 6-12 months for 5 years
Edmonds et al., 2003 [17]	63/F	No trauma. Presentation: Progressive right upper thigh pain	Metastatic solid malignancy	Metastatic colon adenocarcinoma	Radiograph; CT; MRI; chest CT	Management: NR. Outcome: NR
Bonshahi et al., 2004 [18]	Case 1: 90/F	Fall backward twisting left leg. Presentation: Unable to mobilize due to pain	Benign/nonpathologic	Osteoporotic traumatic LT fracture; no tumor on postmortem	X-ray	Management: Initial nonoperative; later DHS after intertrochanteric fracture. Outcome: Died postoperatively of bronchopneumonia/chest complications
Bonshahi et al., 2004 [18]	Case 2: 79/F	Fall backward from stool. Presentation: Unable to bear weight due to pain	Benign/nonpathologic	Osteoporotic traumatic LT fracture	X-ray	Management: Initially full weight-bearing with Zimmer frame; later DHS after intertrochanteric fracture. Outcome: Uneventful postoperative recovery
Bonshahi et al., 2004 [18]	Case 3: 84/F	Slipped on kitchen floor and fell backward. Presentation: Failed mobility assessment; displaced isolated LT fracture	Benign/nonpathologic	Osteoporotic traumatic LT fracture	X-ray	Management: Prophylactic DHS before mobilization. Outcome: Bearing weight with assistance at discharge to rehab day 6
Singh et al., 2015 [19]	40/F	Road traffic accident/dashboard-like injury. Presentation: Severe right groin pain, thigh swelling, unable to perform SLR	Benign/nonpathologic	Nonpathologic displaced LT avulsion	X-ray; skeletal survey; labs; serial x-rays	Management: Derotation boot/bar brace/cast; partial weight-bearing. Outcome: Symptom-free by 6 weeks; no pathology on serial radiographs
Bertin et al., 1984 [20]	Case 1: 28/M	Putting on trousers. Presentation: Left groin pain	Metastatic solid malignancy	Pancreatic islet-cell carcinoma metastasis	Radiograph; bone scan; arteriogram; rib biopsy; GI series; pancreas biopsy	Management: Initial crutches/NWB; radiation/chemotherapy for metastases; later sliding nail after subtrochanteric fracture. Outcome: Subtrochanteric fracture nearly healed after fixation; died Jan 1978 after prolonged hospital course and multiple abdominal surgeries
Bertin et al., 1984 [20]	Case 2: 52/F	No trauma; starting up stairs. Presentation: Right groin pain	Metastatic solid malignancy	Metastatic follicular thyroid carcinoma	Radiograph; bone scan; tomograms	Management: Thyroidectomy + prophylactic sliding nail; radioactive iodine; later radiation; local resection/prosthetic replacement. Outcome: No subtrochanteric fracture before replacement; increasing pain/enlarging lesion at 2 years
Bertin et al., 1984 [20]	Case 3: NR/NR	Slight/no trauma. Presentation: Hip/groin pain	Metastatic solid malignancy	Prostate cancer metastasis	NR	Management: Initial conservative care; later ORIF after subtrochanteric fracture. Outcome: NR
Bertin et al., 1984 [20]	Case 4: NR/NR	Slight/no trauma. Presentation: Hip/groin pain	Metastatic solid malignancy	Adenocarcinoma of unknown origin metastasis	NR	Management: Initial conservative care; later ORIF after subtrochanteric fracture. Outcome: NR
Heiney and Leeson, 2009 [21]	Late 40s/F	Twisting/slight slip in kitchen; no fall. Presentation: Right hip/groin pain, inability to ambulate	Hematologic malignancy	Recurrent acute myeloid leukemia	Radiograph; CT; bone scan; MRI	Management: Prophylactic IM reconstruction nail; oncology care; palliative radiation. Outcome: Pain-free ambulation 4 months; hospice; died 4 months after surgery
Reategui-Villegas et al., 2013 [22]	69/M	No trauma. Presentation: Right hip pain, worse at night, 2 months	Metastatic solid malignancy	Non-small cell lung carcinoma	Hip x-ray; chest x-ray; bronchoscopy; tumor markers; bone scintigraphy; CT	Management: Prophylactic IM nail and chemo planned but not performed. Outcome: Died of respiratory failure 19 days after hospitalization
Phan and Vu, 2018 [23]	58/M	Fall onto buttocks. Presentation: Acute right hip pain and low back pain	Metastatic solid malignancy	Nasopharyngeal carcinoma metastasis; nondifferentiated carcinoma	Pelvic x-ray; CT; MRI	Management: Refused further treatment; discharged. Outcome: NR
Uzun et al., 2016 [24]	86/M	Leg stuck at door; staggered, no fall. Presentation: Right hip pain and difficulty walking	Benign/nonpathologic	Displaced LT fracture without malignancy	Radiograph; CT; labs	Management: Cable and plate fixation. Outcome: Painless hip mobility/no walking difficulty at 3 months
Koçer et al., 2021 [25]	55/M	Atraumatic; standing from bus seat. Presentation: Right hip pain/limp; later dyspnea/chest pain	Metastatic solid malignancy	Metastatic left perihilar bronchial carcinoma	X-ray; CT pelvis; chest CT; bronchoscopy; PET	Management: Initial conservative care; later TFN-A osteosynthesis/cementoplasty followed by radiotherapy. Outcome: No pain and walking without assistance; PET showed no recurrence by Oct 2020
Phillips et al., 1988 [26]	Case 1: 55/M	No significant trauma. Presentation: Right hip pain	Metastatic solid malignancy	Colon adenocarcinoma	Radiograph; specimen radiograph/autopsy confirmation noted in figures	Management: NR. Outcome: NR
Phillips et al., 1988 [26]	Case 2: 38/M	No significant trauma. Presentation: Right hip and groin pain; chest x-ray - large mass	Metastatic solid malignancy	Large-cell carcinoma	Radiograph; CT; radionuclide bone scan; chest X-ray	Management: NR. Outcome: NR
Phillips et al., 1988 [26]	Case 3: 64/M	No significant trauma. Presentation: Left hip pain	Metastatic solid malignancy	Prostate adenocarcinoma	Radiograph	Management: NR. Outcome: NR
Phillips et al., 1988 [26]	Case 4: 51/M	No significant trauma. Presentation: Right hip and upper leg pain	Hematologic malignancy	Non-Hodgkin lymphoma	Radiograph; MRI	Management: NR. Outcome: NR
Gradwohl and Mailliard, 1987 [27]	61/M	Severe cough after aspiration while drinking wine. Presentation: Sudden knife-like right groin pain	Metastatic solid malignancy	History of esophageal squamous cell carcinoma; bone scan suspicious for right rib and right mid-femur metastases	X-ray; bone scan; prior CT/bone scan surveillance	Management: Orthopedic management NR. Outcome: NR after LT fracture
Papineni et al., 2025 [28]	31/M	Minor motorcycle accident (~10 km/h fall onto left side). Presentation: Left hip pain/tenderness with flexion and rotation; concurrent bilateral olecranon avulsion fractures	Benign/nonpathologic	Renal osteodystrophy with secondary hyperparathyroidism in end-stage renal disease; left iliopsoas/LT avulsion	Pelvis radiographs; CT pelvis; MRI hip; laboratory assessment including calcium, phosphate, PTH, alkaline phosphatase, creatinine/eGFR	Management: LT fracture treated conservatively; left olecranon surgically repaired. Outcome: LT-specific follow-up NR

Etiologic Distribution

**Figure 2:**
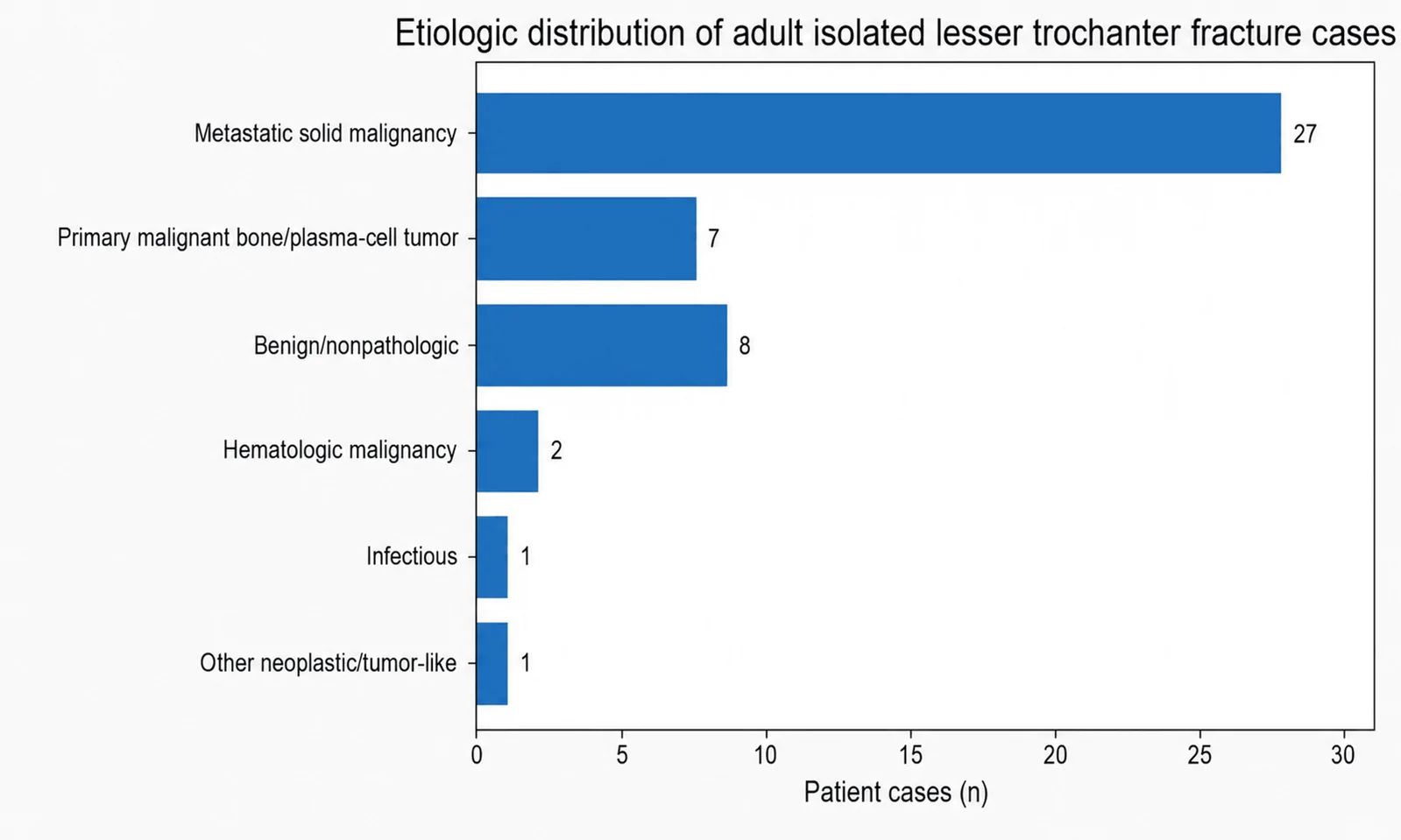
Etiologic distribution of adult isolated lesser trochanter fracture cases Patient-level case counts are based on extracted original/new cases only (N = 46).

Table 5 and Figure 2 show the etiologic distribution. Metastatic solid malignancy made up the largest group, accounting for 27 of 46 cases (58.7%). Primary malignant bone or plasma-cell tumors accounted for seven cases (15.2%), and benign/nonpathologic etiologies, including renal osteodystrophy, accounted for eight cases (17.4%). Hematologic malignancy was reported in two cases (4.3%), while infectious disease and other neoplastic/tumor-like disease each accounted for one case (2.2%).

**Table 5:** Etiologic distribution of adult isolated lesser trochanter fracture cases Studies and patient counts are based on extracted original/new cases only. Individual studies may contribute cases to more than one etiologic group; therefore, study counts across categories are not mutually exclusive and do not sum to 28.

Etiology group	Studies with cases (n)	Patient cases (n)	% of patient cases	Representative diagnoses
Metastatic solid malignancy	17	27	58.7%	Lung, renal cell, breast, colon, thyroid, prostate, paraganglioma, esophageal, pancreatic/islet cells
Primary malignant bone/plasma-cell tumor	4	7	15.2%	Osteosarcoma, Ewing sarcoma, chondrosarcoma, solitary plasmacytoma
Benign/nonpathologic	6	8	17.4%	Stress/repetitive-use, traumatic/athletic, osteoporotic/elderly, renal osteodystrophy/secondary hyperparathyroidism
Hematologic malignancy	2	2	4.3%	Acute myeloid leukemia, non-Hodgkin lymphoma
Infectious	1	1	2.2%	Mycobacterium tuberculosis
Other neoplastic/tumor-like	1	1	2.2%	Langerhans cell histiocytosis

Diagnostic Workup and Management

Table 6 summarizes diagnostic workup and treatment. Atraumatic, nontraumatic, or no significant trauma was explicitly documented in 27 of 46 cases (58.7%). Among the 36 malignant cases, malignancy was first recognized after the LT fracture in 25 cases (25/36, 69.4%; also 25/46, 54.3% of all included cases), was known before the fracture in eight cases (8/36, 22.2%; 8/46, 17.4% overall), and was unclear or not reported in three cases (3/36, 8.3%; 3/46, 6.5% overall). Radiographs were documented in 41 of 46 cases (89.1%), CT in 30 cases (65.2%), MRI in 23 cases (50.0%), bone scan/scintigraphy in 12 cases (26.1%), and PET or PET-CT in 5 cases (10.9%). LT/proximal femoral lesion tissue diagnosis or pathology confirmation was documented in 18 cases (39.1%); this category required biopsy, operative pathology, or pathologic confirmation of the fracture-associated LT/proximal femoral lesion and did not include prior malignancy history alone. These percentages describe what was reported in the published cases and do not measure diagnostic accuracy, comparative effectiveness, or recommended utilization.

**Table 6:** Diagnostic workup, management, and reporting summary Counts reflect patient-level documentation in the extracted data fields. NR or unavailable details were not assumed to be negative. The tissue diagnosis/pathology variable refers to biopsy, operative specimen pathology, or other pathologic confirmation of the fracture-associated LT/proximal femoral lesion; it does not count a prior cancer diagnosis or remote biopsy of a primary tumor alone. The three malignancy-timing categories are mutually exclusive and account for all 36 malignant cases. Imaging, treatment, and follow-up percentages are reporting frequencies in published cases and should not be interpreted as measures of sensitivity, diagnostic accuracy, comparative effectiveness, or recommended use. LT: Lesser trochanter; NR: Not reported.

Variables	Patient cases with documented feature (n)	% of patient cases	Denominator
Atraumatic/nontraumatic or no significant trauma documented	27	58.7%	46
Known malignancy before LT fracture documented	8	22.2% (17.4% overall)	36 malignant cases (46 overall)
Malignancy first recognized after LT fracture	25	69.4% (54.3% overall)	36 malignant cases (46 overall)
Timing of malignancy unclear or not reported	3	8.3% (6.5% overall)	36 malignant cases (46 overall)
Radiographs/X-ray documented	41	89.1%	46
CT documented	30	65.2%	46
MRI documented	23	50.0%	46
Bone scan/scintigraphy documented	12	26.1%	46
PET/PET-CT documented	5	10.9%	46
Fracture-associated LT/proximal femoral tissue diagnosis/pathology documented	18	39.1%	46
Orthopedic surgery/fixation/reconstruction documented	27	58.7%	46
Radiation therapy documented	12	26.1%	46
Systemic disease therapy documented	14	30.4%	46
Outcome/follow-up reported	27	58.7%	46

Orthopedic surgery, fixation, or reconstruction of the LT/proximal femoral condition was documented in 27 cases (58.7%). Reported surgical strategies included prophylactic intramedullary nailing, sliding hip screw or dynamic hip screw fixation, tumor prosthesis, bipolar hemiarthroplasty, custom proximal femoral endoprosthesis, en bloc resection, and allograft-prosthesis reconstruction [1,3,10,13,18,20,21,24,25]. The Papineni LT fracture was treated conservatively; surgery addressed the concomitant olecranon injury rather than the proximal femur [28]. Radiation therapy was documented in 12 cases (26.1%), systemic disease therapy in 14 cases (30.4%), and outcome or follow-up in 27 cases (58.7%).

Discussion

Malignancy dominated the reported adult LT fracture literature. In the extracted dataset, malignant etiologies accounted for 36 of 46 published cases (78.3%), including 27 metastatic solid tumors. This value is a reporting frequency within a selected case-report and case-series evidence base, not an estimate of the prevalence of malignancy among unselected adults with LT fracture. Publication and selection bias likely favor unusual, severe, and malignant presentations, while uncomplicated benign cases may be underreported. The pattern is nevertheless consistent with earlier radiology reports describing atraumatic LT avulsion as a potential sign of proximal femoral tumor infiltration and supports a low threshold for further evaluation when the mechanism is atraumatic, low energy, unclear, or the radiographic appearance is suspicious [14,20,26,29].

The design-specific JBI appraisal directly informed interpretation of the synthesis. Case reports generally described the presenting condition and diagnostic workup, but adverse events and longitudinal outcomes were frequently absent. In the case series, consecutive and complete inclusion of eligible cases was often unclear, and outcome reporting was inconsistent. These limitations increase selection and reporting bias and reduce representativeness; therefore, the findings are descriptive and hypothesis-generating rather than estimates of prevalence, diagnostic performance, or treatment superiority.

The differential should not stop at malignancy. Benign, infectious, and metabolic diagnoses were also found. Bonshahi et al. described elderly patients with isolated LT fractures in whom occult intertrochanteric instability or osteoporotic injury was clinically relevant [18]. Singh et al. reported a nonpathologic adult LT fracture after trauma [19], and Miyake et al. described an adult stress avulsion related to repetitive baseball pitching [4]. Sharma et al. reported tuberculosis as an infectious mimic, while McCambridge et al. reported Langerhans cell histiocytosis in an 18-year-old [10,16]. Papineni et al. added renal osteodystrophy with secondary hyperparathyroidism in a young adult with end-stage renal disease, expanding the benign/nonpathologic spectrum beyond trauma and osteoporosis [28]. The concurrent olecranon fractures in that report did not alter the anatomical classification of the radiographically isolated LT fracture.

Radiographs are an appropriate starting point, but they are not enough when the presentation is atraumatic, low energy, or clinically discordant. An X-ray may show the fracture while missing marrow replacement, soft-tissue extension, skip lesions, or systemic disease burden. James and Davies highlighted this problem in the setting of initial anteroposterior hip imaging, where tumor infiltration may be subtle and additional projections or MRI can reveal destructive disease [29]. In the present review, CT and MRI were frequently used, and tissue diagnosis/pathology of the fracture-associated LT/proximal femoral lesion was documented in 18 cases (39.1%). MRI helps define local marrow and soft-tissue involvement, while CT, bone scan, and PET/CT can support staging or primary-tumor search. When neoplasm or infection remains in the differential, tissue diagnosis is especially important before definitive oncologic reconstruction.

Management often moves beyond routine fracture care. An isolated LT fragment may be the visible part of a larger proximal femoral lesion that can progress to subtrochanteric or intertrochanteric fracture. Several reports used prophylactic fixation or reconstruction to prevent catastrophic fracture and permit mobilization [1,3,13,18,20,21,25]. Treatment still has to be individualized based on lesion size, expected survival, systemic disease status, tumor histology, functional goals, and patient preference. Benign stress, traumatic, or metabolic cases may recover with conservative treatment and management of the underlying disorder, whereas malignant cases often require orthopedic stabilization, radiation, systemic therapy, or a combination of these approaches.

Suggested Diagnostic Approach

The following is an expert-informed approach based on the included reports and musculoskeletal-oncology guidance; it has not been formally validated by the included studies as a diagnostic pathway. In adults with an atraumatic, low-energy, unclear-mechanism, or radiographically suspicious isolated LT fracture, MRI of the pelvis/proximal femur may be considered after initial radiographs to define marrow and soft-tissue involvement. CT can further characterize cortical destruction and assist procedural planning. When imaging raises concern for destructive disease, infection, or systemic malignancy, staging studies and selected laboratory testing should be individualized rather than applied as a fixed panel [29,33]. Referral to orthopedic oncology should occur before biopsy, prophylactic fixation, or definitive tumor surgery whenever a primary bone tumor or indeterminate destructive lesion remains possible. Biopsy should be coordinated with the orthopedic oncologist, musculoskeletal radiologist, and pathologist so that the tract can be incorporated into any subsequent resection and that contamination of uninvolved compartments is minimized [34]. Clearly traumatic or metabolic cases with reassuring imaging may be managed according to fracture stability, the underlying bone disorder, and patient factors, while malignancy and infection remain in the differential until reasonably excluded.

Limitations

The limitations mainly reflect the source literature. Most reports were case reports or small case series, making the evidence highly susceptible to publication and selection bias; the data cannot establish the true incidence of LT fracture or the prevalence of malignancy in an unselected adult population. Diagnostic workup, follow-up duration, and outcome reporting varied across reports, and the JBI appraisal identified recurrent limitations in longitudinal outcome reporting and in the consecutive or complete inclusion of case-series patients. Several older articles contained limited patient-level detail, and some newer articles included literature-review summaries without uniform data fields. Counts therefore represent explicit documentation in published reports rather than population-level rates, diagnostic accuracy, or comparative effectiveness. The English-language restriction, adopted because reliable translation resources were unavailable, may have introduced language bias and led to missed cases. The review protocol was not prospectively registered, which is an additional limitation, although the eligibility criteria, extraction fields, etiologic categories, and synthesis plan were defined before extraction. Full-text availability and indexing limitations may also have led to missed reports. One additional report was identified during peer review and included, which improved completeness but also illustrates the possibility of residual missed literature. Finally, only original or clearly new cases were extracted from review-style publications to reduce double-counting, so the patient count may differ from prior narrative summaries.

## Conclusions

An isolated LT fracture in an adult should raise concern for underlying pathology, particularly when the mechanism is atraumatic, low energy, unclear, or discordant with the imaging findings. In this systematic review of 28 studies and 46 published patient cases, 36 cases had malignant etiologies. Among those malignant cases, malignancy was first recognized after the fracture in 25 cases (25/36, 69.4%), was known beforehand in eight cases (8/36, 22.2%), and was unclear or not reported in three cases (3/36, 8.3%). These frequencies reflect a selected case-report and case-series literature and do not estimate the prevalence of malignancy among unselected adults. Primary malignant bone tumors, hematologic malignancy, infection, and benign/nonpathologic causes, including renal osteodystrophy, were also identified. Atraumatic, low-energy, or radiographically suspicious cases warrant advanced imaging and coordinated orthopedic-oncology evaluation, with carefully planned biopsy when indicated; clearly metabolic or traumatic cases should be managed according to the underlying disorder and fracture stability. Early recognition may prevent delayed cancer diagnosis, avoid poorly planned biopsy or fixation, guide stabilization decisions, and improve multidisciplinary coordination.

## References

[1] Cho HS, Lee YK, Yoon BH, Park JW, Ha YC, Koo KH (2020). Isolated avulsion fracture of the lesser trochanter in adults. In Vivo.

[2] Herren C, Weber CD, Pishnamaz M, Dienstknecht T, Kobbe P, Hildebrand F, Pape HC (2015). Fracture of the lesser trochanter as a sign of undiagnosed tumor disease in adults. Eur J Med Res.

[3] Afra R, Boardman DL, Kabo JM, Eckardt JJ (1999). Avulsion fracture of the lesser trochanter as a result of a preliminary malignant tumor of bone. A report of four cases. J Bone Joint Surg Am.

[4] Miyake Y, Mitani S, Namba Y, Kikuoka R (2022). An avulsion fracture of the lesser trochanter of the femur with prodromal symptoms in an adult: a case report and review of literature. J Orthop Case Rep.

[5] Agrawal N, Eslami MH, Abou Ali AN, Reitz KM, Sridharan N (2023). Adductor canal syndrome after lesser trochanter avulsion fracture in a 19-year-old. J Vasc Surg Cases Innov Tech.

[6] Yu R, Auerbach MS, Honda NS (2024). Bone metastasis manifested 52 years after resection of an apparently benign paraganglioma: a case report. SAGE Open Med Case Rep.

[7] Creissen A, Rajeev A, Jain K, Banaszkiewicz P (2020). Preliminary presentation of metastatic non-small cell carcinoma of the lung as atraumatic avulsion fracture of the lesser trochanter. Case Rep Oncol.

[8] Kumar P, Agarwal S, Rajnish RK, Kumar V, Jindal K (2017). Isolated spontaneous atraumatic avulsion of lesser trochanter of femur-a pathognomonic sign of malignancy in adults? A case report and review of literature. J Orthop Case Rep.

[9] Pang MQ, Wu FZ, Chen JY, Zhu RT, Jin G, Gong WJ, Jiang HT (2025). Case report: a case of femoral metastatic cancer misdiagnosed as isolated femoral lesser trochanter avulsion fracture. Front Oncol.

[10] Sharma A, Kamboj K, Kumar R, Sud A (2022). Tuberculosis challenging pathognomicity of atraumatic avulsion of the lesser trochanter for malignancy: a rare case report. JBJS Case Connect.

[11] Vanhoenacker C, Myncke J, Vanhoenacker F (2021). Ewing's sarcoma mimicking an avulsion fracture. J Belg Soc Radiol.

[12] Uddin F, Tayara B, Al-Khateeb H (2016). Stage IV primary bronchogenic carcinoma presenting as a lesser trochanteric avulsion fracture. Curr Orthop Pract.

[13] Rouvillain JL, Jawahdou R, Blanco OL, Benchikh-El-Fegoun A, Enkaoua E, Uzel M (2011). Isolated lesser trochanter fracture in adults: an early indicator of tumor infiltration. Orthop Traumatol Surg Res.

[14] Khoury JG, Brandser EA, Found EM Jr, Buckwalter JA (1998). Non-traumatic lesser trochanter avulsion: a report of three cases. Iowa Orthop J.

[15] Fox TP, Lakkol S, Oliver G (2014). Lesser trochanter fracture: the presenting feature of a more sinister pathology. BMJ Case Rep.

[16] McCambridge TM, Papakyrikos C, Levin A (2023). Langerhans cell histiocytosis presenting as a lesser trochanter fracture in an adolescent: a case report. JBJS Case Connect.

[17] Edmonds LD, Ly JQ, Carter MC, Lusk JD (2003). Quiz case. Nontraumatic avulsion of the lesser trochanter secondary to metastatic adenocarcinoma of the colon. Eur J Radiol.

[18] Bonshahi AY, Knowles D, Hodgson SP (2004). Isolated lesser trochanter fractures in elderly--a case for prophylactic DHS fixation. A case series. Injury.

[19] Singh P, Kumar A, Shekhawat V, Singh P (2015). Nonpathological lesser trochanter fracture in adult: case report and brief review of literature. J Clin Diagn Res.

[20] Bertin KC, Horstman J, Coleman SS (1984). Isolated fracture of the lesser trochanter in adults: an initial manifestation of metastatic malignant disease. J Bone Joint Surg Am.

[21] Heiney JP, Leeson MC (2009). Isolated lesser trochanter fracture associated with leukemia. Am J Orthop (Belle Mead NJ).

[22] Reategui-Villegas D, Carrasco PC, Dijeres AZ, Betancourt JB (2013). Isolated fracture of the lesser trochanter as a first manifestation of metastatic lung carcinoma. J Med Cases.

[23] Phan TQ, Vu TT (2018). Isolated fracture of the lesser trochanter—an indicator of malignancy at proximal femoral head: a case report. Orthop Rheumatol.

[24] Uzun E, Çıraklı A, Günay AE, Mutlu M (2016). Trochanter minor avulsion fracture in an old patient: greater care in the diagnosis of hip pain in the elderly. J Emerg Med Case Rep.

[25] Koçer S, Grácio S, Molnar P, Baumgartner L (2021). A non-traumatic avulsion fracture of the lesser trochanter: other diagnosis not to be missed. Praxis (Bern 1994).

[26] Phillips CD, Pope TL Jr, Jones JE, Keats TE, MacMillan RH 3rd (1988). Nontraumatic avulsion of the lesser trochanter: a pathognomonic sign of metastatic disease?. Skeletal Radiol.

[27] Gradwohl JR, Mailliard JA (1987). Cough induced avulsion of the lesser trochanter. Nebr Med J.

[28] Papineni VR, Botchu R, Iyengar KP, Chapala S, Shah AR, Rathinavelu B (2025). Bilateral olecranon and left lesser trochanter avulsion fractures in renal osteodystrophy: a rare case report and comprehensive review. Acta Med Litu.

[29] James SL, Davies AM (2006). Atraumatic avulsion of the lesser trochanter as an indicator of tumour infiltration. Eur Radiol.

[30] Page MJ, McKenzie JE, Bossuyt PM (2021). The PRISMA 2020 statement: an updated guideline for reporting systematic reviews. BMJ.

[31] Haddaway NR, Page MJ, Pritchard CC, McGuinness LA (2022). PRISMA2020: an R package and Shiny app for producing PRISMA 2020-compliant flow diagrams, with interactivity for optimised digital transparency and open synthesis. Campbell Syst Rev.

[32] Moola S, Munn Z, Tufanaru C (2024). Systematic reviews of etiology and risk. JBI Manual for Evidence Synthesis.

[33] Miller BJ (2019). Use of imaging prior to referral to a musculoskeletal oncologist. J Am Acad Orthop Surg.

[34] Trieu J, Sinnathamby M, Di Bella C (2016). Biopsy and the diagnostic evaluation of musculoskeletal tumours: critical but often missed in the 21st century. ANZ J Surg.

